# Effects of Dehydration Treatment on the Quality of Sweet Wine from Hetianhong Grapes at Different Ripeness Levels

**DOI:** 10.3390/foods15193436

**Published:** 2026-09-25

**Authors:** Dexin Yang, Aoran Zhang, Xianghan Cheng, Xingyuan Yang, Qiling Chen

**Affiliations:** 1 College of Food Science and Pharmacy, Xinjiang Agricultural University, Nongda East Road 311, Urumqi 830052, China; 18083248584@163.com (D.Y.); zar20040513@126.com (A.Z.); 2 College of Agriculture, Henan University of Science and Technology, Luoyang 471023, China; chengxianghan@haust.edu.cn

**Keywords:** Hetianhong grape, dehydration, maturity, sweet white wine, volatile aroma compounds, quality

## Abstract

This study systematically evaluated the effects of dehydration treatment (water loss level of 15%, 30%, and 45%) and grape maturity (20 and 24 °Brix) on the quality of sweet white wines produced from Hetianhong, a native table grape variety in Xinjiang that is currently underutilized in deep processing. Dehydration increased the total sugar and total acid concentrations in grape must while also significantly elevating the alcohol content, total acidity, total reducing phenolic equivalents, DPPH radical-scavenging activity, and total volatile flavor compound content in the resulting wines. Specifically, the total volatile content in group A3 was 5.93 times that of the non-dehydrated control group A0, which was prepared from the same raw material. Notably, dehydration promoted the formation of 3-hydroxy-2-butanone, a compound that contributes creamy, nutty, and roasted notes to the wines’ profile. Furthermore, dehydration significantly elevated the concentrations of aldehydes and ketones, and higher alcohols, with the aldehyde/ketone content in group A3 being 8398.11 times that of A0, and in group B3 being 10,787.98 times that of B0. Conversely, dehydration led to a significant reduction in the levels of volatile fatty acids, The impact on volatile phenols is not significant. Dehydration alters the distribution of acetates: ethyl acetate levels increase in the dehydration group, whereas isopentyl acetate levels decrease as the degree of dehydration increases. Sensory evaluation revealed that dehydration enhanced the fruity, rosy, and creamy attributes of the wine. Moreover, grape maturity significantly increased the total reducing phenolic equivalents (B0 was 2.96 times that of A0), the DPPH radical-scavenging activity, and the total content of volatile flavor compounds, including the individual contents of higher alcohols, acetate esters, volatile fatty acids, and terpenes. In addition, PCA and OPLS–DA effectively differentiated dehydrated from non-dehydrated wines. A total of 27 key volatile compounds were identified (OAV > 1), 10 of which had VIP > 1. Notably, eight volatile compounds, including (Z)-3-hexenyl acetate, 3-hydroxy-2-butanone, isoamyl lactate, ethyl isovalerate, n-propanol, isoamyl alcohol, ethyl acetate, and isobutanol, were more abundant in groups A1, A2, and B1. This study provides a theoretical and technical foundation for the processing of the Xinjiang table grape variety Hetianhong.

## 1. Introduction

Xinjiang, located in Northwestern China, represents the country’s foremost grape-growing region in terms of both cultivated area and annual output [1]. Among the numerous *Vitis vinifera* cultivars grown in this area, Hetianhong has gained particular attention due to its adaptation to the Southern Xinjiang climate. This cultivar is predominantly cultivated in the Hetian region and is distinguished by berries that are notably thick-skinned and fleshy, with a high juice yield. The fruit typically attains a sugar concentration above 22 °Brix while maintaining a balanced acidity, resulting in a palatable sweet–sour flavor profile [2]. In addition, the relatively large berry size combined with a robust skin structure confers favorable postharvest storability and transport tolerance. Hetianhong also exhibits marked tolerance to saline–alkali soils [3], a trait that aligns well with the pedoclimatic conditions prevalent across Xinjiang.

Despite these advantageous characteristics, the commercial utilization of Hetianhong grapes remains largely confined to two traditional pathways: raisin manufacturing and the production of Musalais, a regionally specific fermented beverage [4]. Musalais, which originates from Awat County in Xinjiang, is deeply rooted in local culture and is occasionally described as a surviving relic of ancient Chinese vinicultural practices, with origins dating to the Han and Tang dynasties [5]. As the principal grape cultivar used in Musalais production, Hetianhong is historically and culturally intertwined with this traditional product. However, the potential of Hetianhong grapes for producing dehydrated sweet wine has received limited scientific attention.

The Hetian region is characterized by abundant sunshine, low relative humidity, and strong diurnal temperature fluctuations during the postharvest period, conditions that are highly conducive to natural grape dehydration. Postharvest dehydration, also referred to as grape drying or raisining, is a well-established technique for concentrating sugars, acids, and phenolic compounds in grape berries, thereby enhancing the flavor complexity and sweetness of the resulting wine [6]. The composition of dehydrated grapes, and consequently the quality of the wine produced from them, is strongly influenced by the ripeness level at which the grapes are harvested. As grapes ripen, the accumulation of sugars, organic acids, phenolic constituents, and aroma precursors undergoes dynamic changes; these compounds are further concentrated during dehydration, and together they determine the fermentable substrate and the eventual sensory profile of the sweet wine [7,8]. Grape maturity significantly affects the profile of soluble solids, titratable acidity, and volatile compounds, all of which critically contribute to the quality attributes of dehydrated sweet wines [9].

Postharvest dehydration significantly alters berry metabolism and the aroma profile of the resulting wine; however, research in this area has predominantly focused on wine grape cultivars [10,11,12]. Existing studies, which incorporate harvest maturity, are exclusively centered on wine grape varieties, such as Pinot Noir red wine [13] and fortified Malvasia Moscata [14]. To date, no investigations have been conducted on white table grape cultivars, which are characterized by unique attributes including distinct berry morphology, skin-to-pulp ratio, aroma profile, and an anthocyanin-free phenolic composition.

This study aims to evaluate the effects of grape ripeness, dehydration treatment, and their interaction on the quality of sweet white wine produced from the Hetianhong grape variety. A two-factor experimental design was employed, incorporating multiple maturity levels and dehydration degrees. Dehydration was conducted using air-drying equipment designed to replicate the wind and heat conditions of the Hetian region. The chemical composition and volatile aroma profile of the resulting wines were thoroughly characterized. To our knowledge, this is the first systematic assessment of dehydrated sweet white wine production from a white table grape cultivar. The findings are expected to provide a scientific basis for utilizing the advantageous wind and heat resources of the Hetian region in value-added grape processing.

## 2. Materials and Methods

### 2.1. Raw Material Pretreatment

Hetianhong grapes (*Vitis vinifera* L.) were collected in September 2024 from Yutian County, Hetian Prefecture, Xinjiang, China. Approximately 60 vines with uniform growth vigor were selected using a Z-shaped sampling pattern, with border-row vines excluded to minimize edge effect; the selected vines were divided into two groups (~30 vines per group) designated for harvest at target soluble solids contents of 20, and 24 °Brix, respectively. Following the berry enlargement phase, SSC was monitored every 7 days. On each sampling occasion, three replicate samples were collected per group, with each replicate consisting of 100 berries taken from the top, middle, and bottom positions of clusters on both sun-exposed and shaded sides; berries were immediately pressed, and the juice was analyzed for SSC using a handheld digital refractometer (Pocket Refractometer PAL-3, ATAGO Instruments, Tokyo, Japan), as well as the total sugar content and total acid content. The monitoring frequency was increased to daily when SSC approached within 1–2 °Brix of the target value. Grapes from each group were hand-harvested upon reaching the respective target SSC (20 and 24 °Brix) and immediately transported to the laboratory, with damaged or defective berries manually removed to ensure sample uniformity.

The grapes were then dehydrated to three target weight loss levels (15%, 30%, and 45%) using an electric blast drying oven equipped with a forced-air blowing system (101-1BS; Shanghai Hanyu Technology Co., Ltd., Shanghai, China). The initial grape mass for each treatment was adjusted to achieve the target weight loss, ensuring enough dehydrated grapes for three parallel fermentations per treatment (4.5 kg each): 16.5 kg for 15% reduction, 19.5 kg for 30%, and 25.5 kg for 45%. Dehydration occurred at 45 °C with 1 m/s air velocity and 40% humidity, using equipment mimicking Hetian’s wind and heat. Thermal energy and forced air facilitated drying. Post-dehydration, grape SSC reached 23, 29.5, and 33.5 °Brix in the 20 °Brix group, and 26, 31, and 38.5 °Brix in the 24 °Brix group. Non-dehydrated grapes with sugar contents of 20 °Brix and 24 °Brix served as controls for the production of sweet white wine. Following destemming, the grapes were crushed and placed into 5 L stainless-steel fermentation tanks. Subsequently, potassium metabisulfite (Tianjin Fuchen Chemical Reagent Factory (Tianjin, China)) was added at a concentration of 100.00 mg/L, along with pectinase (LAFFORT, Floirac, France) at a concentration of 40.00 mg/L. Only one dehydration run was performed for each treatment level. Three parallel fermentations were subsequently carried out using aliquots from the same dehydrated batch. Therefore, these three fermentations are process replicates rather than independent replicates of the dehydration factor.

The details of the grape samples subjected to each dehydration treatments are shown in Table 1.

### 2.2. Alcoholic Fermentation

Active *Saccharomyces cerevisiae* (BV818, purchased from Angel Yeast Co., Ltd., Yichang, China) was measured at a concentration of 0.20 g/L and subsequently activated in a 1:1 (*v*/*v*) mixture of grape juice and water at 37 °C for a duration of 30 min. The activated yeast culture was then inoculated into the grape juice, ensuring that the temperature differential did not exceed 5 °C, and alcoholic fermentation was conducted at a constant temperature of 16 °C. Due to the increased degree of raisining and the consequent reduction in juice yield, the raisined grapes were not immediately processed for clarified juice fermentation. During the initial fermentation phase, pump-over was conducted twice daily. In the context of raisined wine fermentation, this process serves to homogenize the mixture, prevent localized overheating of the raisined grapes, and facilitate juice extraction. During the active fermentation phase, pump-over was conducted three times daily. The temperature and specific gravity of the wine were recorded every 12 h. The dehydrated treatments underwent a two-day skin contact period before pressing, whereas the non-dehydrated controls were processed without skin contact. Fermentation was subsequently halted at a specific gravity of 1.050 by transferring the wine to a low-temperature environment (0–4 °C) and adding 100 mg/L of potassium sorbate together with 30 mg/L of potassium metabisulfite. In the A3 and B3 treatments, which experienced a 45% weight loss, fermentation ceased naturally. To prevent contamination due to undesirable microorganisms, these wines were also transferred to a 0–4 °C environment and supplemented with 100 mg/L of potassium sorbate and 30 mg/L of potassium metabisulfite. The duration of fermentation was 7, 10, and 14 days for treatments with 15%, 30%, and 45% weight loss, respectively. All samples were subsequently clarified by fining with 1 g/L of polyvinylpolypyrrolidone (PVPP) for 15 days. Each experimental treatment was performed in triplicate to ensure the reproducibility and reliability of the results.

### 2.3. Physicochemical Analysis

The physicochemical parameters of the wines, including ethanol content, reducing sugars, total acidity (expressed as tartrate equivalent), and pH, were evaluated utilizing a Foss WineScan FT120 instrument (Foss, Hillerød, Denmark).

### 2.4. Determination of Total Reducing Phenolic Equivalents

The total reducing phenolic equivalents (TRPE) was determined according to the method described in the reference [15]. To prepare the standard curve for total phenol content, gallic acid (0.5 g, Shanghai Haiyuan Ye Biotechnology Co., Ltd., Shanghai, China) was precisely weighed, vacuum-dried to a constant weight, dissolved in ethanol, and subsequently diluted with water to a final volume of 100 mL. Aliquots of 0, 1.0, 2.0, 3.0, 5.0, and 10.0 mL were transferred into 100 mL volumetric flasks and brought to volume with water. The resulting concentrations of gallic acid in these standard solutions were 0, 50, 100, 150, 250, and 500 mg/L. From each standard solution of varying concentrations, 1.0 mL was transferred into a 100 mL volumetric flask, followed by the addition of 60 mL of deionized water and 5 mL of Folin–Ciocalteu reagent (Beijing Solaibao Technology Co., Ltd., Beijing, China). After 0.5 to 8 min, 15 mL of a 20% Na_2_CO_3_ solution (Tianjin Zhiyuan Chemical Reagent Co., Ltd., Tianjin, China) was added and mixed thoroughly, after which the volume was made up to 100 mL with water. The standard solutions were allowed to stand at 20 °C in the dark for 2 h, after which the absorbance was measured at 765 nm. The resulting standard curve equation was determined to be y = 0.1348*x* − 0.0285, with an R^2^ value of 0.9953.

For sample analysis, an aliquot of wine (1 mL) was transferred into a 100 mL volumetric flask, followed by the addition of 60 mL of distilled water and 5 mL of Folin–Ciocalteu reagent. The subsequent procedures were identical to those described for the construction of the calibration curve, and the absorbance of the resulting solution was measured under the same conditions. The TRPE of the wine samples was calculated according to Equation (1) [15]:

(1) $$X\;=\;\frac{C}{V}\times \;P$$

where X is the TRPE of the wine sample (mg/L); *C* is the TRPE determined in the test solution (µg); *V* is the volume of the liquid to be tested (mL) into the colorimetric tube; and P is the dilution factor of the sample.

### 2.5. Evaluation of Antioxidant Capacity

The DPPH radical-scavenging activity of wine was assessed as follows: 12.5 mg of DPPH (2,2–diphenyl–1–picrylhydrazyl, purchased from Beijing Coolaber Technology Co., Ltd., Beijing, China) was dissolved in methanol and diluted to a total volume of 100 mL. Prior to analysis, this stock solution was further diluted by a factor of five to achieve a final concentration of 25 mg/L to ensure it was freshly prepared for each assay.

For the assay, a 0.1 mL aliquot of the wine sample was mixed with 4.9 mL of the DPPH methanolic solution for a final volume of 5 mL. After thorough mixing, the reaction was allowed to proceed for 30 min in the dark, and the absorbance was then measured at 517 nm (A_i_). To correct for the absorbance contributed by the intrinsic color of the wine itself, a sample blank was prepared in which the DPPH solution was replaced with an equal volume of methanol, and the corresponding absorbance was recorded as A_0_. In addition, a reagent control was prepared by replacing the wine sample with an equal volume of methanol, and its absorbance was recorded as A_Z_. The DPPH radical-scavenging activity was calculated according to the following equation [16]:

DPPH radical-scavenging activity (%) = [1 − (A_i_ − A_0_)/A_Z_] × 100

All analyses were performed in triplicate.

### 2.6. The Determination of the Volatile Flavor Substances

Volatile flavor compounds were analyzed via headspace solid-phase microextraction coupled with gas chromatography–mass spectrometry (HS-SPME-GC-MS, Agilent 8890–7000e) [17]. Totals of 5.0 mL diluted wine sample, 1.0 g NaCl, and 10.0 µL 4-methyl-2-pentanol (1.0 g/L, internal standard) were placed in a 20 mL headspace vial. The vial was equilibrated at 40 °C for 30 min and then extracted at the same temperature under magnetic stirring at 250 rpm for 30 min with a DVB/CAR/PDMS fiber. The fiber was then desorbed in the GC injector at 250 °C for 8 min in split mode (split ratio 5:1). GC separation was carried out using an HP–INNOWAX column (60 m × 250 μm × 0.25 μm) with helium as the carrier gas (1.0 mL/min). The temperature program was as follows: 50 °C (1 min) → 220 °C at 2˚C/min → 280 °C at 5 °C/min, with a post-run at 280 °C for 1 min. For MS conditions, EI was at 70 eV, with *m*/*z* as 30–350; the ion source, transfer line, and quadrupole temperatures were set at 230, 250, and 150 °C, respectively.

Compounds were identified by RI and mass spectra (standards or NIST20). Quantification was performed using the external standard calibration method. Calibration curves were constructed in a simulated wine matrix, with 15 concentration levels prepared for each standard. The simulated wine matrix consisted of 12% (*v*/*v*) ethanol and 6.5 g/L tartaric acid, with the pH adjusted to 3.4. The same matrix was used for sample dilution: wine samples were adjusted to 12% (*v*/*v*) ethanol before GC–MS analysis. For compounds lacking authentic reference standards, semi-quantification was performed using structurally analogous compounds. All samples were analyzed in triplicate, and results are reported as mean values. 

### 2.7. Analysis of Key Flavor Components

The odor activity value (OAV), which is the ratio of the concentration of an individual aroma compound to its odor threshold, was employed to assess the contribution of each compound to the wine’s overall aroma profile [18]. The OAV was determined using Equation (1), expressed as OAV = $\frac{C}{Threshold/(µg/L)}$, where C represents the mass concentration of the aroma compound in micrograms per liter (µg/L).

### 2.8. Quantitative Descriptive Sensory Analysis

Quantitative descriptive analysis (QDA) was performed following the protocol described [19], and the detailed procedures were as follows. All sensory analyses were conducted in the sensory laboratory of Xinjiang Agricultural University, equipped with 40 individual evaluation booths, an independent sample preparation room, and controlled lighting and ventilation. Twenty-five senior enology students (aged 19–24 years, senior undergraduate students majoring in enology with 1–3 years of wine sensory evaluation experience) were initially screened for normal olfactory acuity and descriptive ability in accordance with GB/T 16291.1-2012 [20], and subsequently underwent a four-week training program (20–30 h) comprising fundamental knowledge of wine, basic sensory ability training, and identification of sensory attributes with the establishment and use of intensity scales. Based on training performance, 18 qualified panelists (8 males, 10 females) were selected as the final descriptive analysis panel. A consensus vocabulary of sensory descriptors was developed by two national Level-1 wine tasters with reference to GB/T 15038-2006, and refined according to the aroma and taste profile of the wine following four principles: typicality, distinctiveness, standardization, and independence. Samples (20 mL) were presented monadically in ISO-compliant wine-tasting glasses coded with random three-digit numbers at 17 °C. To account for temperature-dependent aroma volatility, two aliquots of each sample were provided per evaluation session. Each session was completed within 1 h, and the entire assessment was repeated three times. Panelists rated the intensity of each descriptor on a 10-point scale (1–3, weak; 4–6, moderate; 7–8, strong; 9–10, very strong). Then, the mean scores for each attribute were calculated for subsequent statistical analyses.

### 2.9. Data Analysis

All experiments were conducted in triplicate, with results presented as the mean ± standard deviation (SD). The three parallel fermentations were treated as observational replicates in the ANOVA, but inferences are limited to the specific dehydration batches used in this experiment. Data compilation was carried out using Microsoft Excel 2021, while bar charts were produced utilizing Origin 2019b. OPLS-DA was performed using SIMCA 14.1. The data matrix comprised 24 samples (8 treatments × 3 replicates) and 27 volatile compounds (OAV > 1); each replicate sample was treated as an independent sample. Prior to analysis, the data underwent logarithmic transformation and Pareto scaling. The categorical labels were assigned based on maturity (A vs. B) and degree of dehydration (0, 1, 2, 3). The model was validated using 7-fold cross-validation and additionally tested with 200 permutation tests. The paper reports various statistical measures for the model, including R^2^X, R^2^Y, and Q^2^. Two-way analysis of variance (two-way ANOVA) was performed to evaluate the effects of the two factors and their interaction on the measured indicators. When interaction effects were significant, an independent samples *t*-test was employed to conduct a simple effect analysis comparing the maturity levels of A and B at the same water loss rate. Before conducting the *t*-test, normality and homogeneity of variance were checked; if the variances were homogeneous, Student's *t*-test was used; if the variances were heterogeneous, Welch’s *t*-test was applied. A *p*-value < 0.05 indicates a statistically significant difference. All statistical analyses were conducted using IBM SPSS Statistics version 26.0. software.

## 3. Results and Discussion

### 3.1. Physicochemical Parameters of Grapes and Wines Under Different Ripeness and Dehydration Treatments

Key physicochemical parameters of wine samples produced from Hetianhong grapes at two maturity stages under different dehydration treatments were measured, and the results are presented in Table 1 and Table 2.

Table 1 shows that the dehydration treatment of grapes significantly increased the sugar and acid content of the grapes. For grapes harvested at two distinct maturity levels, a dehydration level of 45% resulted in a 1.4-fold increase in total sugar content. Additionally, in raw material with initial soluble solids of 24 °Brix, a 45% dehydration level led to an increase in total acid content by 1.35 g/L. The fermentation outcomes demonstrated that the substantial rise in the total sugar content following dehydration treatment contributed to a marked elevation in the alcohol content of the resulting sweet wine. Furthermore, for both raw materials subjected to 45% dehydration, the synergistic effect of the alcohol generated during fermentation and the residual sugar in the wine inhibited further alcoholic fermentation by yeast, culminating in the natural cessation of the fermentation process. The visual characteristics of wines subjected to various treatments are illustrated in Figure 1. As depicted, the yellow–brown hues of the wines intensify progressively with increasing maturity and dehydration levels. Notably, the alcohol content in wines under treatments A2, A3, B2, and B3 exceeded 15% (*v*/*v*).

Alcoholic fermentation markedly decreased the total acid content in the wine; however, wines produced from dehydrated grapes exhibited a significantly higher total acid content compared to those made from non-dehydrated grapes. In addition, the comparative analysis of groups A and B demonstrated that during the desiccation process of grape berries, the titratable acidity (TA) initially declined, followed by a marginal increase in the later stages. This fluctuation is likely due to the ongoing respiratory activity of freshly harvested grape berries [6]. In the early phase of drying, elevated temperatures enhanced both gluconeogenesis and respiration, leading to a reduction in malic acid content and, consequently, a decrease in total titratable acidity [21]. As the drying process advanced, aerobic respiration in the grape berries ceased [22], halting the conversion of malic acid; at this juncture, the continuous evaporation of water resulted in the concentration of solutes, causing a slight recovery in titratable acid content. Moreover, the potential occurrence of the Fenton reaction during drying may oxidize tartaric acid to glyoxylic acid, thereby increasing the pH of dried grape berries compared to fresh grapes, which aligns with previous research findings [23,24]. The same titratable acidity can correspond to either a low or high pH in different samples; this is because the pH depends on the concentration of free protons and the dissociation equilibrium, whereas the TA depends on the total number of titratable protons. The exchange of K^+^ and H^+^ ions in grape berries leads to the substitution of some acidic protons by metal cations; these protons no longer contribute to the total acidity, yet the pH is still governed by free protons. During the dehydration process, water evaporation leads to the concentration of metal cations such as K^+^, an increase in the extent of exchange, and a decrease in the proportion of free protons; as a result, the pH may rise. In contrast, TA reflects the total amount of titratable protons and is indirectly influenced by the concentration of K^+^; therefore, the direction of its change may differ [25]. The volatile acid content in all wine samples was below 1.2 g/L; however, it increased significantly with the degree of dehydration. This suggests that a higher proportion of solids correlates with elevated volatile acid levels. Among all treatment groups, the B3 group demonstrated the highest total sugar content in the grape must, resulting in wine that displayed significantly elevated levels of residual sugar, ethanol content, and volatile acid.

### 3.2. Maturity and Dehydration Significantly Enhances the Total Reducing Phenolic Equivalents in Hetianhong Wines

Phenolic compounds contribute significantly to the antioxidant capacity of white wine and are primarily classified into flavonoids and non-flavonoids [26,27]. Non-flavonoids, which are colorless, represent the predominant components of phenolic compounds in white wine, including phenolic acids and volatile phenols. These compounds play a crucial role in defining the sensory characteristics and quality of white wines. Phenolic acids, such as hydroxycinnamic and hydroxybenzoic acids, are particularly significant due to their influence on the wine’s stability and oxidative properties [28,29]. Figure 2 (left) illustrates the total phenolic content, expressed as total reducing phenolic equivalents (TRPE), of each sample. Dehydration resulted in a statistically significant increase in the TRPE of the wines (*p* < 0.05). Specifically, the TRPE of sample B3 was 8.19 times that of B0 (the same raw material without dehydration treatment) and 2.05 times that of B1 (the same raw material with 15% dehydration). Similarly, the TRPE of A3 was 8.67 times that of A0 and 2.18 times that of A1. Grape maturity also significantly increased the TRPE of the wines (*p* < 0.05). In particular, the TRPE of B0 was 2.96 times that of A0 revealed the maturity level greatly affects the TRPE of wines.

Furthermore, wines produced from dehydrated grapes generally exhibited a higher TRPE compared to those from non-dehydrated grapes [30]. This is primarily attributed to the fermentation process, which involved maceration on the grape skins for two days prior to pressing, coupled with the increased skin-to-juice ratio in dehydrated grapes that facilitated phenolic extraction [31]. The maturity of the grapes significantly enhanced the TRPE of the wine, as the accumulation of phenolic compounds per berry continues post-veraison and is subsequently released during maceration [32].

### 3.3. Maturity and Dehydration Significantly Enhances the Antioxidant Capacity in Hetianhong Wines

The antioxidant capacity of white wine is primarily attributed to its phenolic compound content [33]. The experiment employed DPPH radical-scavenging assay to assess the wine’s antioxidant capacity (Figure 2 (right)). In alignment with the trend observed in TRPE, an increase in the maturity and degree of dehydration of the raw material significantly enhanced the antioxidant capacity of Hetianhong wines (*p* < 0.05). A two-way ANOVA was employed to examine the effects of maturity, water loss rate, and their interaction on DPPH. The results revealed that the main effect of maturity was highly significant (F = 105.75, df = 1, *p* < 0.001), the main effect of water loss rate was highly significant (F = 1843.94, df = 3, *p* < 0.001), and the interaction between maturity and water loss rate was also highly significant (F = 93.40, df = 3, *p* < 0.001). Due to the significant interaction, separate one-way ANOVA analyses and Tukey’s HSD post hoc tests were conducted for each level of maturity. At maturity level A, the order of DPPH values was A3 > A2 > A1 > A0, and the differences among the water loss rates were significant; at maturity level B, B3 was significantly higher than B1, B2, and B0, whereas no significant difference was observed between B1 and B2. At the same water loss rates (0%,15%, and 30%), the DPPH values for maturity level B were significantly higher than those for maturity level A; however, at a water loss rate of 45%, no significant difference was observed between the two levels. Among all the samples, group B3 demonstrated the highest antioxidant capacity, which was 1.26 times greater than that of B0. Furthermore, group A3 exhibited an antioxidant capacity that was 1.48 times greater than that of A0 and 1.21 times greater than that of A1. Consistent with other studies, dehydration treatment significantly increased the DPPH radical-scavenging capacity, and this finding corroborates the report by [34] that postharvest dehydration of grapes enhances their antioxidant capacity.

### 3.4. Impact of Maturity and Dehydration on Hetianhong Wine Flavor Volatiles

Volatile aroma compounds are crucial in defining the characteristic flavor profile of wine [35,36]. Through the application of headspace solid-phase microextraction coupled with gas chromatography–mass spectrometry (HS-SPME-GC-MS), a total of 58 volatile flavor compounds were identified (see Supplementary Tables S1 and S2). These compounds included 11 higher alcohols, 31 esters (including 15 ethyl esters, seven acetate esters, and nine other esters), seven fatty acids, two aldehydes and ketones, five aromatic phenols, and three terpenes.

Dehydration significantly increased the total amount of volatile flavor compounds in the wine samples (*p* < 0.05, Figure 3). Compared to non-dehydrated wines (A0, B0), the total volatile compound content of dehydrated wines increased substantially, with the A3 group reaching 5.93 times that of A0. Dehydration significantly elevated the total contents of aldehydes and ketones, acetate esters, and higher alcohols; in particular, the total aldehyde and ketone content of the A3 group was 8398.11 times that of A0, and that of the B3 group was 10,787.98 times that of B0. 2,4-Dibutylphenol is a widely reported pollutant originating from plastics, pipelines, and SPME/GC consumables; after removing this pollutant, dehydration has no significant effect on the total volatile phenols content. Volatile fatty acids exhibit a significant downward trend as the drying degree increases. Notably, 3-hydroxy-2-butanone was detected only in dehydrated wines, with the highest content observed in the A2 treatment group; this compound may impart a creamy flavor and rose fragrance to the wine [37,38].

### 3.5. Key Differential Compounds and Odor Activity Value Analysis

Further analysis revealed that 27 volatile flavor compounds exhibited OAV > 1 (Table 3) [18]. And the clustering heatmap (Figure 4) indicated that samples A0 and B0 were predominantly characterized by hexanoic acid, octanoic acid, and isovaleric acid. In contrast, samples A3, B2, and B3 were primarily associated with isobutyric acid and n-butanol, contributing phenolic, chemical, fatty, and fusel alcohol aromas. Meanwhile, the A1, A2, and B1 groups were characterized by the accumulation of isoamyl acetate, ethyl butyrate, methyl decanoate, ethyl acetate, isoamyl alcohol, n-propanol, (Z)-3-hexenyl acetate, 3-hydroxy-2-butanone, and isobutanol, contributing banana, sweet fruit, sour fruit, strawberry, fruity, wine-like, fruity-sweet, alcoholic, pungent, and bitter aromas.

To further elucidate the overall differences among the eight treatments (comprising two maturity levels and four dehydration degrees, with three replicates per treatment), OPLS-DA was conducted on the 27 volatile compound. The resulting score plot (Figure 5a,b) delineated three distinct clusters: samples A1, A2, and B1 were located in the second quadrant; samples A3, B2, and B3 in the third quadrant; and samples A0 and B0 in the fourth quadrant. A total of 10 key differential compounds were identified using OPLS-DA, with Variable Importance in Projection (VIP) values exceeding one and a significance threshold of *p* < 0.01, indicating their significance in distinguishing among different groups (see Figure 5c). The model demonstrated excellent fit and predictive capability, evidenced by R^2^X(cum) = 0.946, R^2^Y(cum) = 0.949, and Q^2^ = 0.881. A 200-permutation test validated the model’s robustness, showing no overfitting, as the Q^2^ regression line intersected the y-axis below zero (Figure 5d). Correspondingly, the identified compounds (VIP > 1 and *p* < 0.01) include (Z)-3-hexenyl acetate, 3-hydroxy-2-butanone (characterized by creamy and buttery notes), isoamyl lactate (noted for creamy, nutty, and fruity attributes), ethyl isovalerate (associated with banana and sweet fruit aromas), n-propanol, isoamyl alcohol (described as alcoholic, pungent, and bitter), ethyl acetate (fruity and sweet), isobutanol (fusel-like), isobutyric acid (phenol, chemical smell, fat), and n-butanol (medicinal and alcoholic). Of these, the first eight compounds were predominantly abundant in groups A1, A2, and B1, while the latter two were enriched in groups A3, B2, and B3.

### 3.6. Impact of Maturity and Dehydration on Hetianhong Wine Sensory Quality

Sensory analysis results (Figure 6) showed that dehydration level and maturity had a decisive influence on the sensory profile of the wines [43]. Dehydrated wines exhibited the highest intensities of sweet fruit, rose flower, creamy, and caramel attributes, whereas vegetable and sour fruit notes were less pronounced, and the caramel and floral intensities increased progressively with dehydration level. In contrast, non-dehydrated wines displayed green, sour fruit, and fresh floral notes reminiscent of plant greenness and refreshing fruitiness, but lacked the creamy and caramel attributes that confer fullness, leaving the alcoholic burning sensation unmasked and the overall profile thinner and less complex. With increasing maturity, the proportions of sweet fruit and floral notes increased, while the green vegetable perception declined, yielding a softer wine in which the residual vegetal note was balanced by fruity sweetness and overall acceptability improved.

The observed sensory differences were chemically aligned with the volatile profiles. The sweet-fruit and creamy attributes of the A1, A2, and B1 dehydrated wines were linked to the accumulation of ethyl acetate (fruity and sweet), 3-hydroxy-2-butanone (buttery, creamy), and isoamyl lactate (creamy, nutty, and fruity attributes); all three were identified as key differential markers of this cluster (VIP > 1, *p* < 0.01; Figure 5c), constituting the fruity-sweet and dairy-like backbone of these wines, while the rose-floral note was consistent with the high OAV of phenethyl acetate (pleasant floral). In contrast, the non-dehydrated controls (A0, B0) were characterized by a predominance of hexanoic, octanoic, and isovaleric acids, resulting in a sharp, pungent-sour character with a lack of the volatiles that contribute to fullness, thereby failing to mask the pungency of ethanol. In the A3, B2, and B3 groups of dehydrated wines, ester-derived sweetness was accompanied by isobutyric acid (phenol, chemical smell, fat) and n-butanol (medicinal and alcoholic). Collectively, the integration of OAV, orthogonal partial least squares discriminant analysis (OPLS-DA), and sensory data suggests that moderate dehydration, when combined with appropriate maturity, optimally balances aroma richness with freshness. However, several of these sensory differences were not statistically significant.

## 4. Conclusions

This study demonstrated that harvest maturity and postharvest dehydration jointly modulate the flavor and antioxidant quality of sweet wines produced from Hetianhong, a native Xinjiang table grape traditionally utilized for fresh consumption and Musalais winemaking. The dehydration process progressively enhanced the total reducing phenolic equivalents and DPPH radical-scavenging activity, while also altering the volatile profile of the wine. Specifically, there was a substantial accumulation of aldehydes, ketones, acetate esters, and higher alcohols, alongside the novel formation of 3-hydroxy-2-butanone, a compound contributing creamy and rose fragrance notes. Conversely, individual volatile phenols exhibited no significant change, while volatile fatty acids exhibited a decrease.

Harvesting at advanced maturity stages significantly enhanced the total reducing phenolic content and the accumulation of volatile compounds, thereby amplifying sweet fruit and rose aromas while diminishing green or vegetative notes. The integration of higher maturity levels with an optimal degree of dehydration resulted in wines exhibiting intensified aromas of sweet fruit, rose, cream, and caramel. Using the criteria of OAV > 1 and VIP > 1, we identified 10 key differential aroma compounds. Among these, isobutyric acid and n-butanol were associated with the 45% dehydrated samples, whereas the remaining eight compounds were characteristic of the 30% and 15% dehydrated samples, as well as the non-dehydrated wines. Collectively, these findings offer a theoretical foundation and technical guidance for the value-added processing of Hetianhong grapes and the development of high-value sweet wines from indigenous varieties.

Nonetheless, it is important to recognize several limitations inherent in this study. Firstly, the dehydration process was conducted under laboratory conditions designed to simulate the hot, dry wind environment characteristic of Hetian. These controlled conditions are unable to fully replicate the diurnal variations in temperature, humidity, and airflow experienced during traditional open-air sun-drying. Furthermore, the grapes utilized in this study were obtained from a single vintage and growing region. Future research should aim to compare simulated dehydration with natural field drying and on-vine withering while also extending the analysis across multiple vintages and terroirs. Finally, only one independent dehydration batch was produced for each treatment level, and the three parallel fermentations were derived from the same batch. Therefore, the statistical inference regarding the dehydration effect should be interpreted with caution, and future studies with independent dehydration replicates are needed to validate the findings. In summary, this study provides a theoretical and technical framework for the value-added processing of Hetianhong grapes and the effective utilization of Hetian’s abundant light–thermal resources.

## Figures and Tables

**Figure 1:**
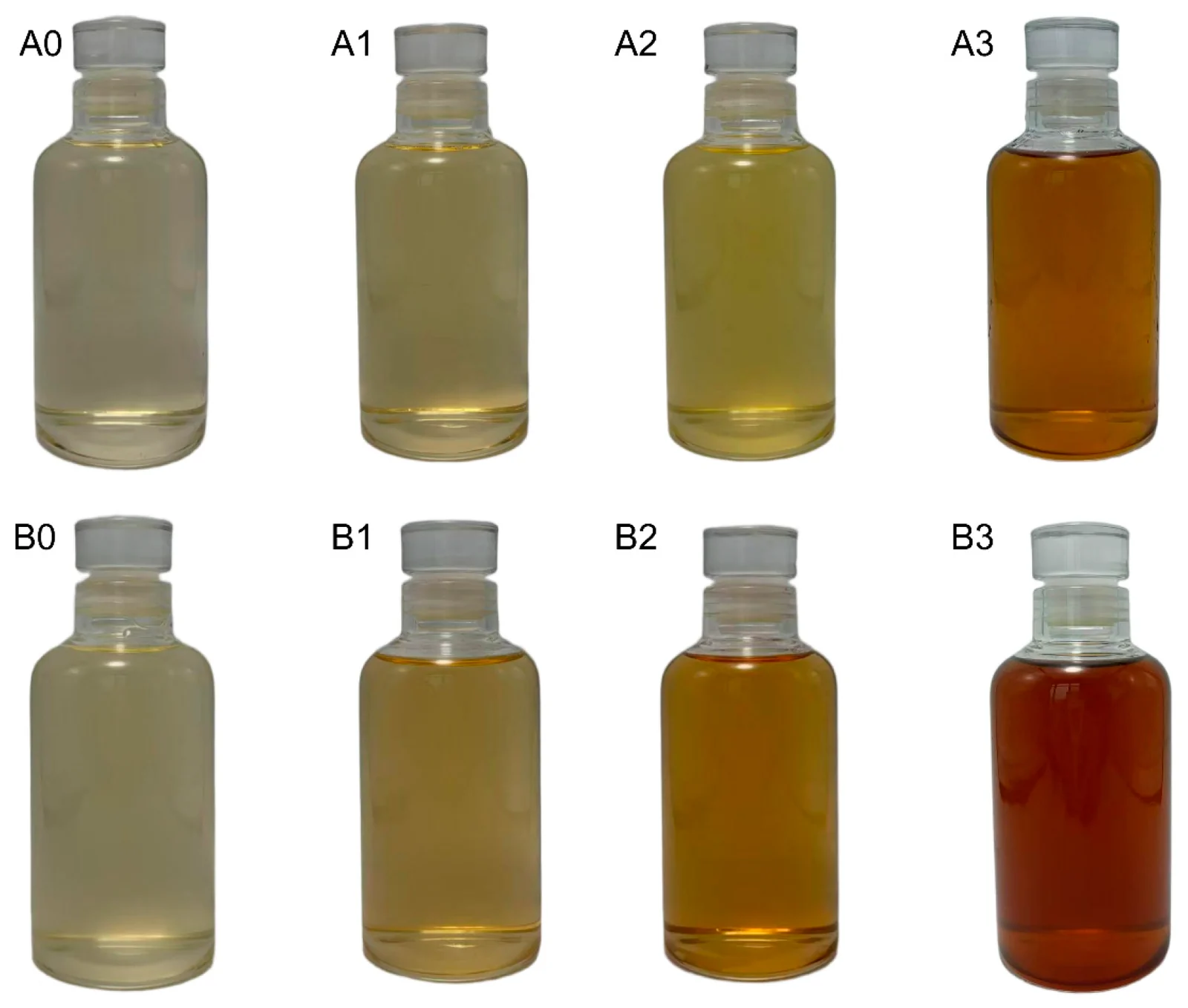
Photographs of wine samples following different processing conditions.

**Figure 2:**
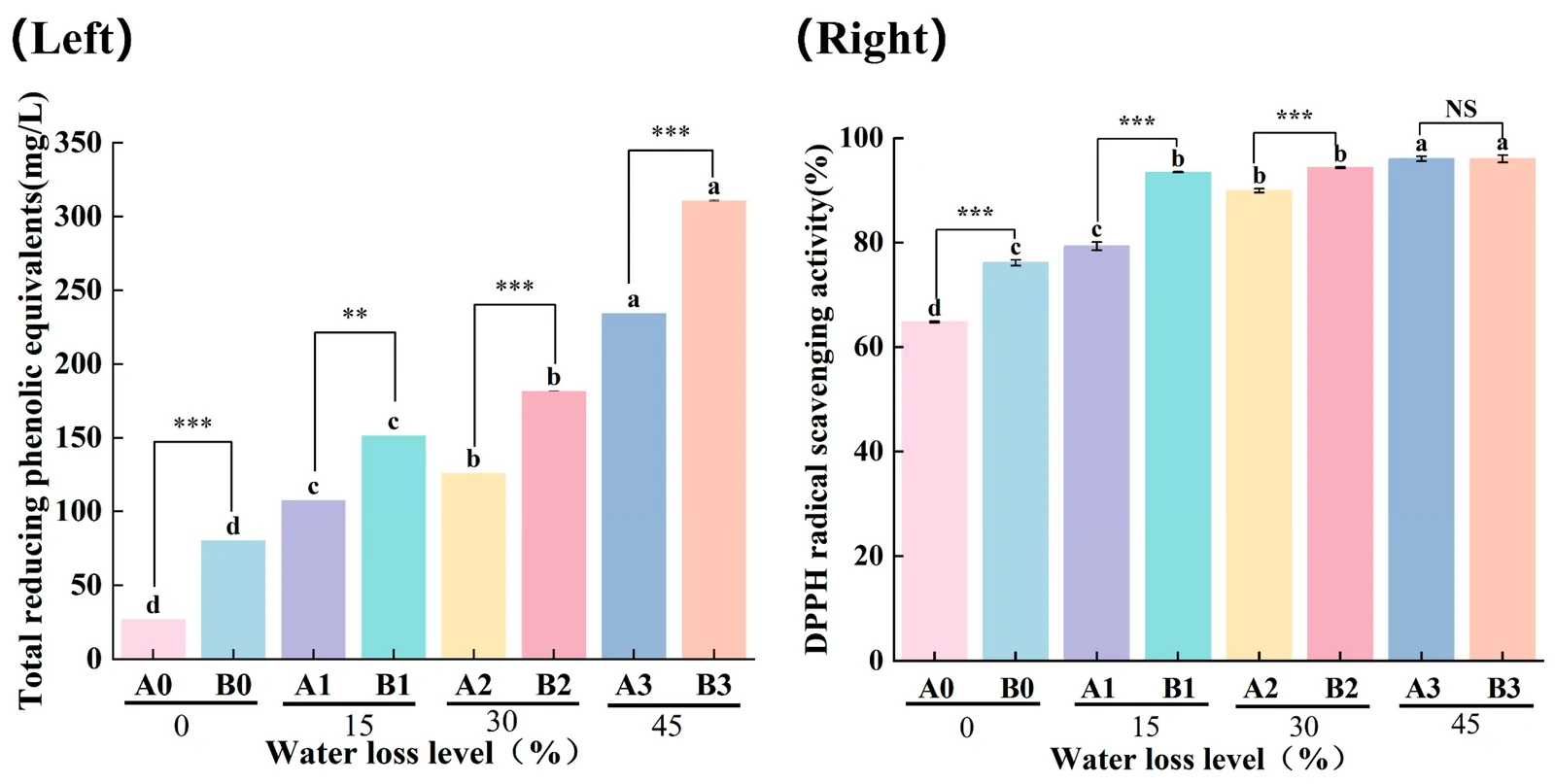
Changes in total reducing phenolics under varying maturity levels and water loss rates (**left**) and DPPH in Hetianhong sweet white wine across various maturity stages and levels of dehydration (**right**). Data are presented as mean ± standard deviation. Different lowercase letters indicate significant differences among different water loss rates at the same ripening stage (Tukey HSD, *p* < 0.05). Stars denote differences between ripening stages A and B at the same water loss rate: NS indicates no significant difference (*p* ≥ 0.05); ** *p* < 0.01; *** *p* < 0.001 (independent samples *t*-test).

**Figure 3:**
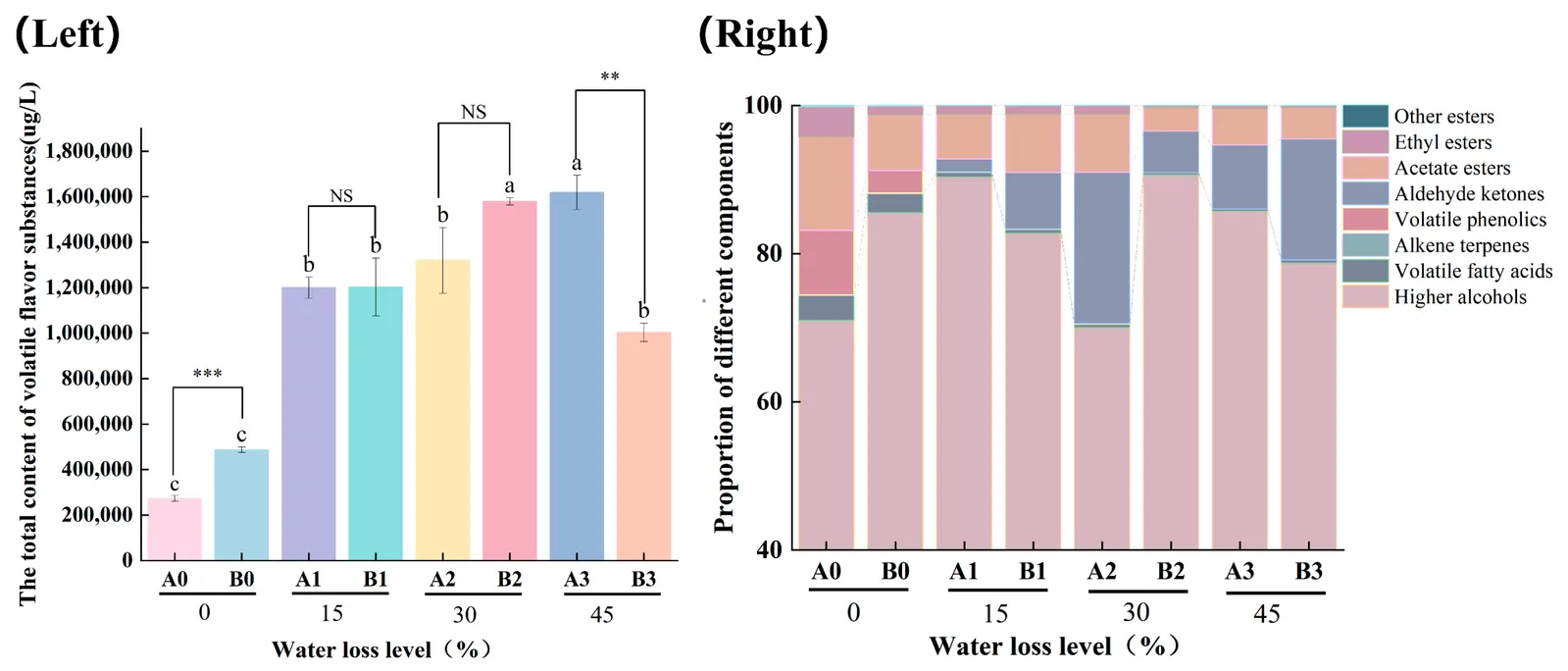
Effects of maturity and dehydration levels on volatile flavor compound total content in Hetianhong wines. Data are presented as mean ± standard deviation. Different lowercase letters indicate significant differences among different water loss rates at the same ripening stage (Tukey HSD, *p* < 0.05). Stars denote differences between ripening stages A and B at the same water loss rate: NS indicates no significant difference (*p* ≥ 0.05); ** p < 0.01; *** *p* < 0.001 (independent samples *t*-test). The bar chart displays the total content of aroma compounds in different groups and in red wine (**Left**). Stacked chart illustrating the total content of different classes of aroma substances in the Hetianhong wines. Each class is distinguished by a unique color (**Right**).

**Figure 4:**
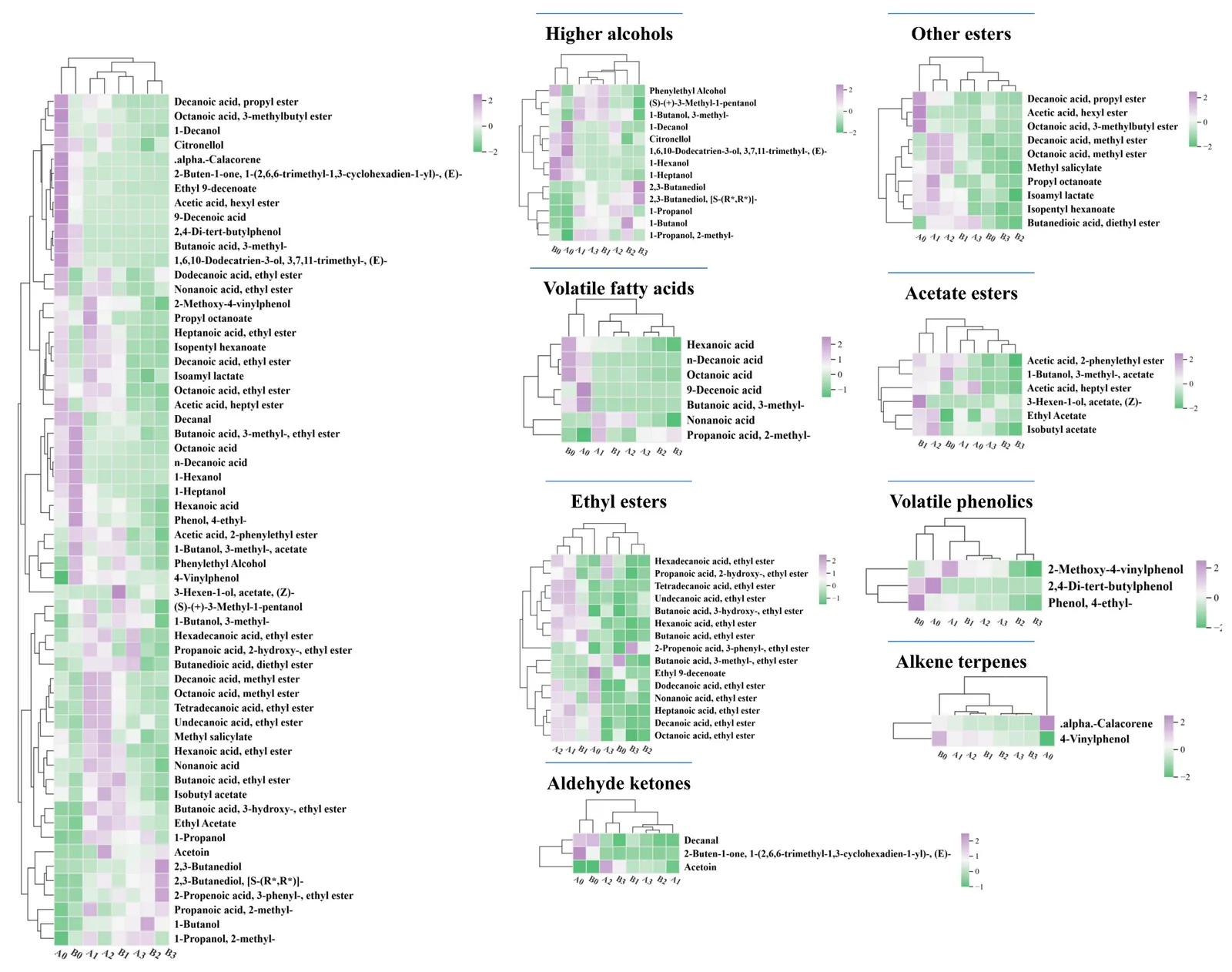
OAV heat map of key aroma-active compounds in wine samples at different maturity stages and dehydration levels (OAV ≥ 1).

**Figure 5:**
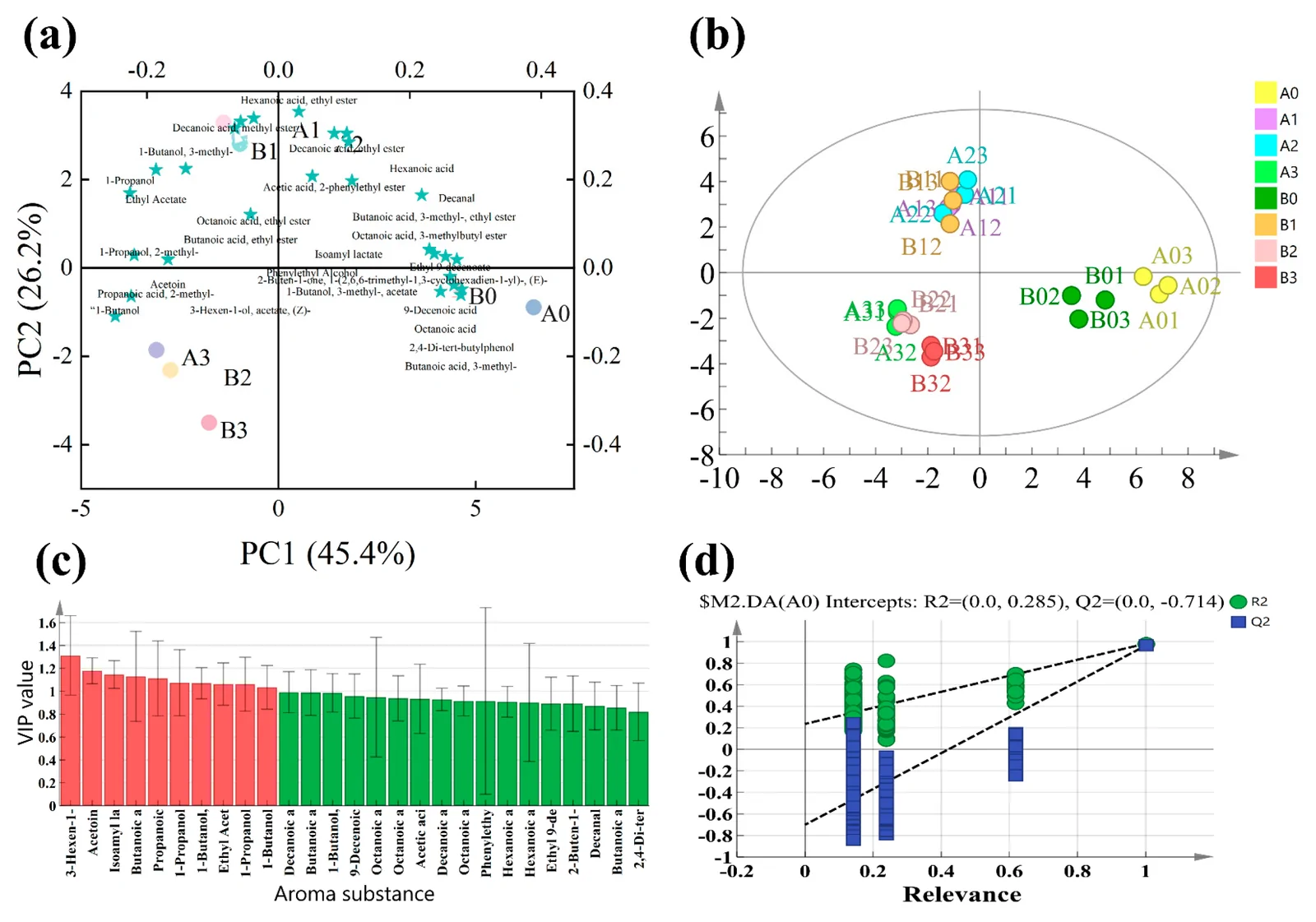
Results of orthogonal partial least squares discriminant analysis (OPLS-DA) of aroma components in different wine groups. Note: (**a**) PCA; (**b**) OPLS-DA score plot; (**c**) variable importance in projection (VIP) scores of volatile compounds derived from the OPLS-DA model; (**d**) permutation test (*n* = 200) validating model reliability.

**Figure 6:**
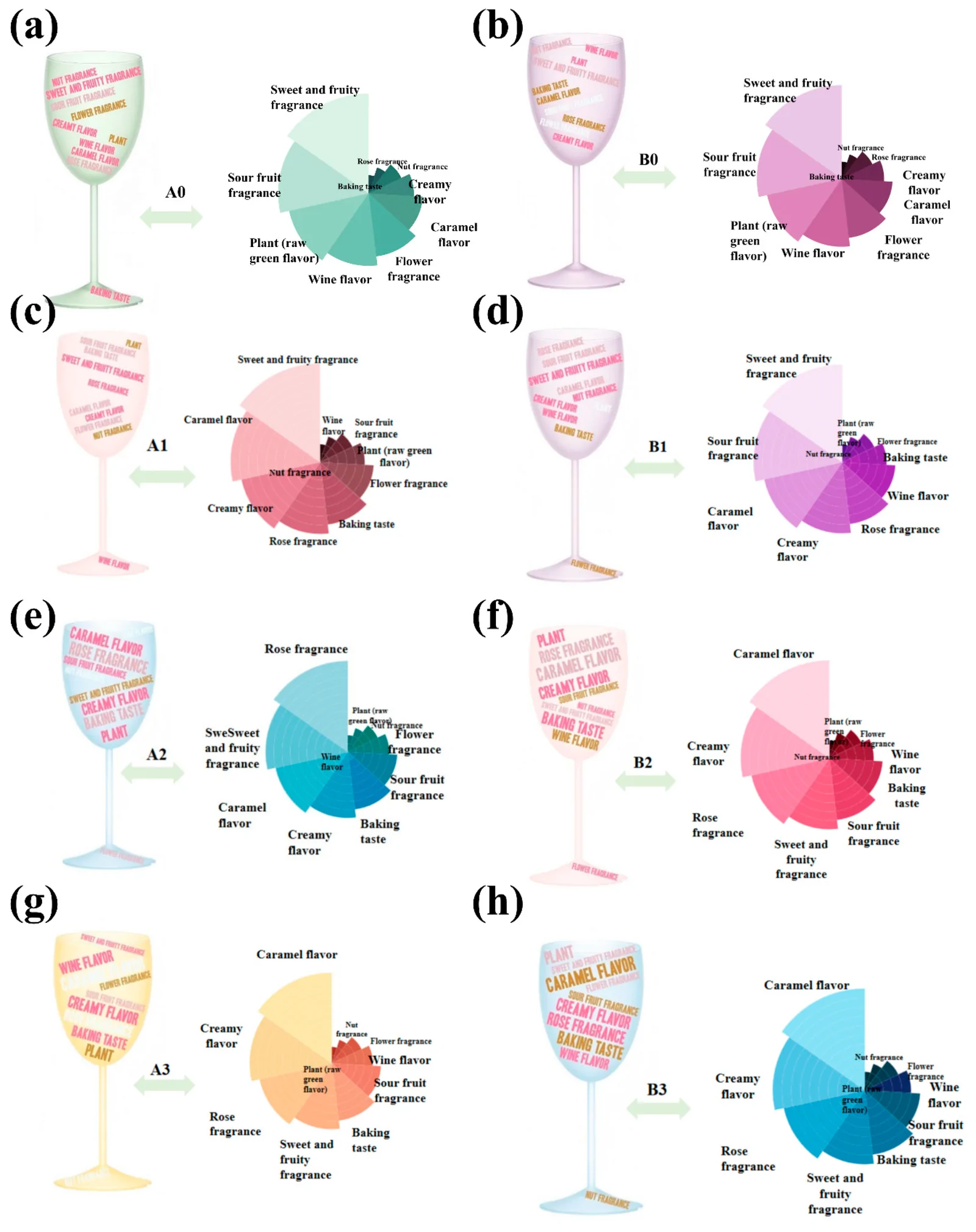
Nightingale rose diagram for wines from different groups. Note: (**a**) A0 group aroma rose diagram; (**b**) B0 group aroma rose diagram; (**c**) A1 group aroma rose diagram; (**d**) B1 group aroma rose diagram; (**e**) A2 group aroma rose diagram; (**f**) B2 group aroma rose diagram; (**g**) A3 group aroma rose diagram; (**h**) B3 group aroma rose diagram.

**Table 1:** Dehydration treatments of raw grape materials.

Group	Fresh Grape			Water Loss Level %	Dehydration Grape	
	Soluble Solids Content (°Brix)	Total Sugar (g/L)	Total Acid (g/L)		Total Sugar (g/L)	Total Acid (g/L)
A0	20	216.7 ± 0.1 ^a^	8.46 ± 00 ^a^	0	-	-
A1	20			15	226.5 ± 0.21 ^c^	11.1 ± 0.2 ^b^
A2	20			30	278.5 ± 0.31 ^b^	11.5 ± 0.1 ^a^
A3	20			45	308.5 ± 0.2 ^a^	10.5 ± 0.2 ^c^
B0	24	255.3 ± 0.6 ^b^	7.15 ± 0.06 ^b^	0	-	-
B1	24			15	238.23 ± 0.25 ^c^	7.47 ± 0.15 ^c^
B2	24			30	308.13 ± 0.15 ^b^	8.07 ± 0.21 ^b^
B3	24			45	362.2 ± 0.3 ^a^	8.5 ± 0.1 ^a^

Note: Values are means ± standard deviation (*n* = 3). For fresh grapes (0% dehydration), A0 and B0 were compared using an independent-samples *t*-test. For dehydrated grapes (15%, 30%, 45%), data were analyzed by two-way ANOVA with maturity and dehydration level as fixed factors, followed by Tukey’s HSD test. Different lowercase letters within the same column indicate significant differences among treatments at *p* < 0.05. Fresh grape values are shown for reference and were not included in the two-way ANOVA, as the 0% level does not exist for dehydrated grapes.

**Table 2:** The impact of drying and dehydration treatments on the fundamental physicochemical properties of wine.

Group	Alcohol Content (% vol)	Reducing Sugar (g/L)	Total Acid (g/L)	pH	Volatile Acid (g/L)
A0	8.01 ± 0.03 ^g^	46.9 ± 0.1 ^f^	6.27 ± 0.25 ^e^	3.47 ± 0.02 ^h^	0.4 ± 0.01 ^c^
A1	11.05 ± 0.03 ^e^	46.2 ± 0.63 ^f^	8.07 ± 0.06 ^b^	3.85 ± 0.05 ^e^	0.53 ± 0.06 ^c^
A2	15.06 ± 0.06 ^c^	48.1 ± 1.36 ^e^	7.63 ± 0.15 ^c^	4.04 ± 0.03 ^d^	0.7 ± 0.01 ^b^
A3	15.86 ± 0.04 ^b^	97.37 ± 0.42 ^b^	9.7 ± 0.2 ^a^	3.57 ± 0.02 ^g^	0.8 ± 0.02 ^ab^
B0	10.04 ± 0.03 ^f^	52.63 ± 0.21 ^d^	5.8 ± 0.2 ^f^	3.77 ± 0.02 ^f^	0.4 ± 0.01 ^c^
B1	12.72 ± 0.04 ^d^	53.1 ± 0.32 ^cd^	6.77 ± 0.2 ^d^	4.16 ± 0.01 ^c^	0.7 ± 0.01 ^b^
B2	15.85 ± 0.05 ^b^	53.87 ± 0.21 ^c^	7.9 ± 0.1 ^bc^	4.53 ± 0.04 ^b^	0.77 ± 0.12 ^ab^
B3	16.13 ± 0.04 ^a^	99.33 ± 0.91 ^a^	7.57 ± 0.32 ^c^	4.66 ± 0.03 ^a^	0.9 ± 0.01 ^a^

Note: Total acidity (TA) was quantified using Foss WineScan FT120 instrument (Foss, Hillerød, Denmark) as a tartrate equivalent (g/L). Volatile acidity is expressed in terms of acetic acid (g/L). Different lowercase letters indicate statistically significant differences between groups (*p* < 0.05) (the same applies below). Values are means ± standard deviation (*n* = 3). Different lowercase letters within the same column indicate significant differences among the eight treatments at *p* < 0.05, based on two-way ANOVA followed by Tukey’s HSD post hoc test. Treatments sharing the same letter are not significantly different.

**Table 3:** Key aromatic components and their odor activity values in Hetianhong wines at different maturity stages and dehydration levels.

**CAS**	Name	Category	RI	Odor Activity Values of Key Flavor Compounds								Threshold (µg/L)	Reference	Sensory Descriptors
				A0	A1	A2	A3	B0	B1	B2	B3			
71-23-8	1-Propanol	D1	1055	0.446	6.784	6.322	4.993	0.912	4.270	6.175	2.116	50,000	[39]	
78-83-1	2-Methyl-1-propanol	D2	1116	0.743	1.613	1.092	1.789	1.176	1.481	1.605	1.12	40,000	[39]	Mixed and mellow fragrance
71-36-3	1-Butanol	D3	1169	0.145	1.929	1.899	4.079	0.640	2.380	4.618	2.346	150,000	[39]	Medicinal flavor, mellow fragrance
123-51-3	3-Methyl-1-butanol	D4	1228	3.473	11.1	6.873	8.771	6.524	9.828	8.414	2.829	30,000	[39]	Mellow, stimulating, bitter
60-12-8	Phenylethyl Alcohol	D12	1942	1.310	2.411	1.863	1.597	3.268	2.964	1.632	1.272	10,000	[40]	Flower fragrance, pollen smell
141-78-6	Ethyl Acetate	E1	916	3.968	8.771	12.3	10.15	2.590	11.06	6.219	5.587	7500	[39]	Fruity, sweet
105-54-4	Ethyl butyrate	F1	1057	163.7	274.4	339.8	167.6	74.06	406.6	101.6	62.72	20	[39]	Sour fruit fragrance, strawberry, fruity fragrance
108-64-5	Ethyl isovalerate	F2	1087	0.844	0.392	0.485	0.488	1.277	0.453	0.138	0.232	3	[40]	Banana, sweet fruit
123-92-2	Isoamyl acetate	E3	1137	134.02	200.98	361.86	109.03	586.23	370.66	117.093	14.958	30	[41]	Banana, sweet fruit
123-66-0	Ethyl caproate	F3	1249	4.86	6.8	7.93	1.73	2.43	6.35	1.64	0.94	140	[40]	Fruit
3681-71-8	Leaf acetate	E4	1336	1.647	0.313	0.288	0.141	0.401	23.95	5.405	1.652	13		
106-32-1	Ethyl caprylate	F6	1455	1409.8	1426.65	1466.39	234.933	987.75	929.25	237.31	116.91	2	[39]	Pineapple, pear, flower fragrance
19329-89-6	Propanoic acid,2-hydroxy-, 3-methylbutyl ester	G4	1576	0.971	1.059	0.927	0.777	0.853	0.923	0.704	0.815	200	[41]	Cream, nuts, fruity
110-42-9	Methyl n-caprate	G5	1618	21.26	22.51	22.39	21.12	20.87	21.59	21.03	21.11	4.3	[42]	Wine
110-38-3	Ethyl caprate	F9	1662	8.619	9.038	8.896	1.693	3.564	6.459	2.171	1.897	200	[39]	Fruity fragrance, fat
2035-99-6	Octanoic acid, 3-methylbutyl ester	G7	1681	1.174	0.334	0.23	0.052	0.234	0.224	0.076	0.062	125		Fruity fragrance, cheese
67233-91-4	Ethyl 9-decenoate	F10	1717	15.86	2.187	2.993	0.964	6.071	1.357	1.164	0.917	100	[42]	
103-45-7	Phenethyl acetate	E6	1851	0.956	1.782	1.467	0.498	1.992	2.012	0.818	0.144	250	[41]	Comfortable floral fragrance
513-86-0	3-Hydroxy-2-butanone	H1	1319	ND	62.98	818.5	423.2	ND	277.8	267.1	496.1	329	[41]	Cream, rose fragrance
23726-93-4	beta-Damascenone	H2	1852	70.09	2.996	3.314	0.208	27.81	3.734	0.684	ND	0.05	[39]	Bark, canned peach
112-31-2	Decanal	H3	1508	1.308	0.374	0.599	0.415	1.374	0.715	0.377	0.324	10		Sweet orange, rose flavor
79-31-2	Isobutyric acid	I1	1592	0.514	1.338	0.719	1.378	0.714	0.843	1.062	1.164	2300	[39]	Phenol, chemical smell, fat
503-74-2	Isovaleric acid	I2	1694	15.96	2.804	2.748	2.772	11.25	2.799	2.776	2.734	33.4		Cheese, fruit flavor
142-62-1	Hexanoic acid	I3	1869	5.659	5.05	4.311	1.708	8.725	4.981	2.465	1.609	420	[40]	Cheese, rotten taste
124-07-2	Octanoic acid	I4	2064	8.233	3.013	2.944	0.677	13.1	2.424	1.015	0.701	500	[39]	Cheese, astringent, corrupt
14436-32-9	9-Decenoic acid	I7	2240	5.895	1.093	1.136	1.054	2.088	1.056	1.057	1.054	100		
96-76-4	2,4-Di-tert-butylphenol	K3	2273	118.4	5.037	7.298	1.83	76.48	4.025	1.895	1.242	200	[40]	Phenol flavor

## Data Availability

The dataset used in this study is available upon request from the authors.

## Supplementary Materials

The following supporting information can be downloaded at: https://www.mdpi.com/article/10.3390/foods15193436/s1, Table S1: The total content of various volatile flavor compounds in wine samples at different maturity stages and dehydration levels; Table S2: ANOVA results.
