# Supplementary material for: Effects of Dehydration Treatment on the Quality of Sweet Wine from Hetianhong Grapes at Different Ripeness Levels
Source: Foods. 2026 Sep 25;15(19):3436. doi: 10.3390/foods15193436 (PMC13664371; doi:10.3390/foods15193436)
Supplement: Supplementary file 1 [file foods-15-03436-s001.zip › Supplementary Tables.pdf]

Table S1. The total content of various volatile flavor compounds in wine samples at different maturity stages and dehydration levels.

No.	Compound	CAS No.	SigmaProductNo.	Quantitative Reference Sample	Standard Curve	R^2	Quantitative Type
Higher alcohols							
1	1-Propanol	71-23-8	96566	1-butanol	y=94738.51x+446.79	0.995	analogous compound
2	2-Methyl-1-propanol (isobutanol)	78-83-1	82059	isobutanol	y=47191.92x+24.82	0.999	Orthodox Standard
3	1-Butanol	71-36-3	19422	1-butanol	y=33618.97x-1.99	0.999	Orthodox Standard
4	3-Methyl-1-butanol (isoamylol)	123-51-3	59092	isopentanol	y=16399.33x-32.35	0.999	Orthodox Standard
5	1-Pentanol	71-41-0	77597	1-pentanol	y=7880.64x+0.91	0.998	Orthodox Standard
6	4-Methyl-1-pentanol	626-89-1	M66951	4-methyl-1-pentanol	y=2509.48x-0.63	0.999	Orthodox Standard
7	3-Methyl-1-pentanol	589-35-5	W376205	isobutanol	y=47191.92x+24.82	0.999	analogous compound
8	1-Hexanol	111-27-3	73117	1-hexanol	y=2252.06x-22.86	0.999	Orthodox Standard
9	(E)-2-Hexen-1-ol	928-95-0	W256230	(E)-2-hexen-1-ol	y=3084.5x-133.0	0.979	Orthodox Standard
10	(Z)-2-Hexen-1-ol	928-94-9	224707	(Z)-2-hexen-1-ol	y=19218.34x-0.22	0.994	Orthodox Standard
11	(E)-3-Hexen-1-ol	928-97-2	W435600	(E)-3-hexen-1-ol	y=5483.50x-1.03	0.995	Orthodox Standard
12	(Z)-3-Hexen-1-ol	928-96-1	H12900	(Z)-3-hexen-1-ol	y=7028.63x-5.82	0.999	Orthodox Standard
13	1-Heptanol	111-70-6	72954	1-heptanol	y=23.08x-0.24	0.982	Orthodox Standard
14	2-Ethyl-1-hexanol	104-76-7	8607	2-ethyl-1-hexanol	y=149.68x-1.06	0.999	Orthodox Standard
15	1-Octanol	111-87-5	95446	1-octanol	y=79.90x-3.38	0.996	Orthodox Standard
16	1-Octen-3-ol	3391-86-4	W280518	1-octen-3-ol	y=279.68x-1.20	0.981	Orthodox Standard
17	1-Decanol	112-30-1	239763	1-decanol	y=339.31x-2.17	0.992	Orthodox Standard
18	Benzyl alcohol	100-51-6	8421	benzyl alcohol	y=7786.08x+7.14	0.999	Orthodox Standard
19	2-Phenylethanol	1960/12/8	77861	2-phenylethanol	y=2645.21x-17.88	0.999	Orthodox Standard
20	3-Methylthio-1-propanol (methionol)	505-10-2	W341509	1-pentanol	y=7880.64x+0.91	0.998	analogous compound
21	2,3-Butanediol	513-85-9	B84904	le-vo-/meso-23-butanediol	y=2650700.29x+120.87	0.988	Orthodox Standard
22	Linalool	78-70-6	L2602	linalool	y=192.95x-0.17	0.992	Orthodox Standard
23	α-Terpineol	98-55-5	4899	α-terpineol	y=291.36x-0.14	0.998	Orthodox Standard
24	Citronellol	106-22-9	C83201	citronellol	y=237.08x+0.19	0.997	Orthodox Standard
25	Nerol	106-25-2	268909	nerol	y=212.81x+0.39	0.989	Orthodox Standard
26	Geraniol	106-24-1	48798	geraniol	y=933.52x+1.94	0.99	Orthodox Standard
27	Farnesol	4602-84-0	F203	—	—	—	
Esters (No. 28–50)							
28	Ethyl acetate	141-78-6	319902	—	—	—	
29	Ethyl butyrate	105-54-4	75563	ethyl butanoate	y=1086.68x+5.19	0.999	Orthodox Standard

30	Ethyl isobutyrate	97-62-1	246085	—	—	—	
31	Ethyl 2-methylbutyrate	7452-79-1	306886	—	—	—	
32	Ethyl 3-methylbutyrate (ethyl isovalerate)	108-64-5	112283	—	—	—	
33	Isobutyl acetate	110-19-0	94823	—	—	—	
34	Isoamyl acetate	123-92-2	79857	isoamyl acetate	y=303.44x-0.28	0.999	Orthodox Standard
35	Ethyl hexanoate	123-66-0	148962	ethyl hexanoate	y=263.66x-341.07	0.994	Orthodox Standard
36	Hexyl acetate	142-92-7	25539	hexyl acetate	y=100.28x-5.97	0.999	Orthodox Standard
37	Ethyl lactate	97-64-3	W244015	ethyl lactate	y=56382.02x+117.63	0.999	Orthodox Standard
38	Ethyl 3-hydroxybutyrate	5405-41-4	55821	ethyl butanoate	y=1086.68x+5.19	0.999	analogous compound
39	Ethyl caprylate (ethyl octanoate)	106-32-1	44879	ethyl octanoate	y=106.83x-167.01	0.996	Orthodox Standard
40	Methyl octanoate	111-11-5	21719	methyl octanoate	y=22.47x+0.14	0.999	Orthodox Standard
41	Ethyl nonanoate	123-29-5	W244740	ethyl nonanoate	y=17.14x+1.03	0.984	Orthodox Standard
42	Isopentyl hexanoate	2198-61-0	W207500	isoamyl hexanoate	y=38.49x+14.87	0.996	Orthodox Standard
43	Ethyl caprate (ethyl decanoate)	110-38-3	148970	ethyl decanoate	y=90.99x+172.99	0.998	Orthodox Standard
44	Diethyl succinate	123-25-1	7429	diethyl succinate	y=1586.48x-244.23	0.994	Orthodox Standard
45	Ethyl laurate	106-33-2	61630	ethyl dodecanoate	y=58.72x+70.38	0.982	Orthodox Standard
46	Geranyl acetate	105-87-3	173495	—	—	—	
47	Phenethyl acetate	103-45-7	73747	phenethyl acetate	y=95.74x+1.23	0.999	Orthodox Standard
48	Methyl salicylate	119-36-8	76631	methyl salicylate	y=88.08x+1.60	0.997	Orthodox Standard
49	Ethyl salicylate	118-61-6	112291	phenethyl acetate	y=34.32x+1.68	0.997	analogous compound
50	Ethyl palmitate	628-97-7	P9009	—	—	—	
Volatile fatty acid (No. 51–55)							
51	Acetic acid	64-19-7	71251	—	—	—	
52	Isobutyric acid	79-31-2	I1754	isobutyric acid	y=84060.17x+26.54	0.997	Orthodox Standard
53	Isovaleric acid	503-74-2	129542	isovaleric acid	y=36450.00x+45.00	0.991	Orthodox Standard
54	Octanoic acid	124-07-2	21639	octanoic acid	y=1105.19x+89.46	0.992	Orthodox Standard
55	Decanoic acid	334-48-5	21409	n-decanoic acid	y=710.12x+73.45	0.981	Orthodox Standard
Aldoketones (No. 56–62)							
56	Octanal	124-13-0	O5608	—	—	—	
57	Nonanal	124-19-6	N30803	nonanal	y=137.71x-10.44	0.987	Orthodox Standard
58	Decanal	112-31-2	D7384	decanal	y=132.65x+6.49	0.994	Orthodox Standard
59	Benzaldehyde	100-52-7	9143	benzaldehyde	y=446.12x-3.49	0.991	Orthodox Standard
60	Furfural	1998/1/1	185914	—	—	—	
61	2,3-Butanedione (diacetyl)	431-03-8	B85307	—	—	—	
62	Acetoin	513-86-0	W200832	acetoin	y=534120x-2735.5	0.981	Orthodox Standard
Deisopentadiene and terpenes (No. 63–67)							
63	β-Damascone	23726-93-4	79172	β-ionone	y=12.28x+0.84	0.994	Orthodox

							Standard
64	β-Ionone	79-77-6	I12603	β-ionone	y=12.28x+0.84	0.994	Orthodox Standard
65	Limonene	5989-27-5	183164	—	—	—	
66	Rose oxide	16409-43-1	83915	cis-/trans-rose oxide	y=66.83x-0.001 / y=56.37x+0.029	0.992 / 0.999	Orthodox Standard
67	Whiskey lactone	39212-23-2	W380300	ethyl butanoate	y=1086.68x+5.19	0.999	analogous compound
Phenolic compounds and pyrazine compounds (No. 68–71)							
68	Phenol	108-95-2	1002060250	phenol	y=2211.20x-0.17	0.999	Orthodox Standard
69	4-Ethylphenol	123-07-9	E44205	4-ethyl phenol	y=444.45x+1.44	0.991	Orthodox Standard
70	4-Ethyl-2-methoxyphenol (4-ethylguaiacol)	2785-89-9	W243604	—	—	—	
71	2-Methoxy-3-isobutylpyrazine	24683-00-9	W313203	2-methoxy-3-isobutylpyrazine	y=58.86x-0.20	0.996	Orthodox Standard

Table S2. ANOVA results.

Dependent variable	Source	df	F	p
Total sugar	Maturity	1	360.3	<0.001
	Dehydration	3	8087.6	<0.001
	Maturity × Dehydration	3	16.17	<0.001
Total acid	Maturity	1	123.15	<0.001
	Dehydration	3	174.74	<0.001
	Maturity × Dehydration	3	40.20	<0.001
pH	Maturity	1	1997.3	<0.001
	Dehydration	3	530.4	<0.001
	Maturity × Dehydration	3	231.0	<0.001
Volatile acid	Maturity	1	18.33	<0.001
	Dehydration	3	93.92	<0.001
	Maturity × Dehydration	3	3.16	≈0.055
Alcohol content	Maturity	1	4998.0	<0.001
	Dehydration	3	37656.9	<0.001
	Maturity × Dehydration	3	571.4	<0.001
total reducing phenolic	Maturity	1	44161	<0.0001
	Dehydration	3	78726.9	<0.0001
	Maturity × Dehydration	3	1283	<0.0001
DPPH	Maturity	1	105.75	1.14×10^{-9}
	Dehydration	3	1843.94	$< 2 \times 10^{-16}$
	Maturity × Dehydration	3	93.40	1.21×10^{-12}
